# Clinicopathological characterization and prognostic implication of FOXP3 and CK19 expression in papillary thyroid carcinoma and concomitant Hashimoto's thyroiditis

**DOI:** 10.1038/s41598-020-67615-0

**Published:** 2020-06-30

**Authors:** Salem Youssef Mohamed, Taiseer R. Ibrahim, Samah S. Elbasateeny, Lobna A. Abdelaziz, Shaimaa Farouk, Mahmoud Abdou Yassin, Ahmed Embaby

**Affiliations:** 1 Pathology Department, Faculty of Medicine, Zagazig University, Zagazig, Egypt; 2 Clinical Oncology and Nuclear Medicine Department, Faculty of Medicine, Zagazig University, Zagazig, Egypt; 3 General Surgery Department, Faculty of Medicine, Zagazig University, Zagazig, Egypt; 4 Internal Medicine Department, Faculty of Medicine, Zagazig University, Zagazig, Egypt

**Keywords:** Biochemistry, Cancer, Molecular biology, Biomarkers, Endocrinology, Molecular medicine, Oncology

## Abstract

Papillary thyroid carcinoma (PTC) is considered the most prevalent thyroid malignancy. The association between Hashimoto’s thyroiditis (HT) and PTC is still unclear. We aimed to examine the clinicopathological impact of immunohistochemical staining of FOXP3 and Cytokeratin 19 in PTC and concomitant HT and their correlation with patients' outcome and survival. Eighty thyroid biopsies obtained from patients with PTC were immunostained by FOXP3 and CK19.The patients were treated by radioactive iodine (I^131^) and followed up. FOXP3 and CK19 expression were detected in 45% and 80% studied cases of PTC respectively. 16.7% of PTC with associated HT showed FOXP3+ lymphocytes in lymphocytic infiltrate of HT, while most of PTC associated HT express cytoplasmic CK19 positive Hurtle cells. FOXP3 was more expressed in PTC female patients more than 45 years with higher stage, lymph node, and distant metastasis, extracapsular extension, number of I^131^doses, and cumulative radioiodine doses with a highly statistically significant difference (p < 0.001). The relation was significant between CK19 immunostaining as regard 10-year Overall Survival and death (p value = 0.027 and 0.036, respectively). HT represents a step in the process of autoimmune inflammatory disease ending by the evolution of PTC with better prognosis, therefore appropriate follow up of these cases is needed. FOXP3 tends to be more expressed in PTC cases with worse prognostic variables and is predictable to become a recent prognostic and targeted therapy for PTC. There was a significant relation between CK19 immunostaining and 10 year overall survival.

## Introduction

Hashimoto’s thyroiditis (HT) is the commonest autoimmune disease of the thyroid gland, it is characterized by lymphocytic cellular infiltrate, with the destruction of the thyroid acini and marked fibrosis^1^. It was known for the first time in 1912 by Hakaru Hashimoto, a famous pathologist in Japan^2^. HT is more common in females with the female-to-male ratio of 10:1^3^.

In the past decades, there is a dramatic increase in the incidence of thyroid cancer, and this increase is mainly seen in papillary thyroid carcinoma (PTC) which is more prevalent in females, representing 75–80% of all thyroid cancers^4^. Recently the diagnosis of PTC is increasing all over the world and multiple factors are claimed. Increased use of high-resolution ultrasonography (US) on the thyroid gland and US-guided needle aspiration biopsy (FNAB) and accessible histopathological investigation of surgical biopsies may be responsible for this increase^5^.

Despite the emerging evidence of a link with chemical pollution^6^, ionizing radiation is considered the only environmental factor showing a cause-effect relationship with thyroid cancer^7^. It is noted that lymphocytic infiltration is commonly present in PTC, suggesting immunological mechanisms in the development of PTC^8^.

There is a close clinical relationship between PTC and HT^8,9^. Chronic inflammation is one of the risk factors for cancer pathogenesis especially in thyroid carcinoma^10^.

There are many shreds of evidence of the inflammatory role in carcinogenesis through direct relationship by allowing a suitable environment which is an important element in the majority of cancers. Additionally, the significant mutation rate is more common in tissues with chronic inflammation than in normal ones^11^.

Inflammatory mediators produced by cancer cells may lead to tumor progression through different methods as, increasing cell division, inhibition of apoptosis, activation of angiogenesis, and remodeling of the stroma or suppressing the protective antitumor immunity. Chronic inflammation causes oxidative stress, which leading to DNA damage and genetic alterations such as oncogene expression. Thyroid cancers especially PTC have an inflammatory component that is composed of different types of white blood cells^12^.

Regulatory T cells (Treg) belong to T helper (CD4+) cells which have a vital role in the immune system by suppressing autoimmune reactions through antagonizing the actions of T cells. The transcription factor forkhead box (FOXP3) is essential for this regulation. FOXP3 is a definite biomarker of regulatory T cells (Treg) and important for the genesis and function of these cells^13^.

Forkhead box P3 (FOXP3) transcription factor is one of the forkhead box (FOX) family, characterized by a forkhead domain (FKH) that bound to deoxyribonucleic acid (DNA) playing a role in the control of Treg cells. The loss or alteration of the FOXP3 function leads to a decrease in the number of these cells. Inflammation increases the number of these cells and this considered an important mechanism in protecting malignant cells from the immune system^14^. Increased Treg cells have been detected in the serum of patients with PTC, which in line with this suggestion^15^.

Cytokeratin polypeptide 19 (CK19) is detected with simple and stratified epithelial cells and demonstrated in a strong and diffuse positivity in malignant thyroid tumors; separating them from benign thyroid lesions so it can be used in the diagnosis of these cancers. CK19 importance lies in its increased sensitivity for PTC so the absence of CK19 expression denies the diagnosis of PTC^16^.

The current work aimed to evaluate the clinicopathological significance of immunohistochemical staining of FOXP3 and CK19 in PTC and concomitant HT and their relation to patients' outcome and survival.

## Patients and methods

### Patients and clinical data

A cross-sectional study conducted in the Department of Pathology, Clinical Oncology and Nuclear Medicine, and internal medicine departments, Faculty of Medicine, Zagazig University, Egypt. Eighty Formalin-fixed, paraffin-embedded thyroid biopsy were selected randomly from the archive of the Pathology Department in the period from May 2009 to May 2019. All study participants provided informed written consent before study enrollment. This study was prepared and revised according to the STROBE statement and checklist. Moreover, this study was conducted according to the declaration of Helsinki. The corresponding hematoxylin–eosin slides were reviewed to confirm the diagnosis. The clinicopathological characteristics of our patients including Demographic information, age, gender, tumor stage, pathological variants, non-neoplastic thyroid tissue, the presence of extracapsular extension or multifocality, bilaterality were obtained from the patient’s medical records at Clinical Oncology and Nuclear Medicine. According to the patients' files, the patients underwent surgical resection (total or near-total thyroidectomy) and then referred to the National Cancer Institute, Cairo to receive radioactive iodine therapy according to their risk stratification group based on American Thyroid Association Guidelines (ATA)^17^ and followed up. The data of radioiodine treatment and the patients' outcomes were taken from the patient’s relevant sheets in NCI Cairo. PTC cases showed lymphoplasmacytic infiltration with the formation of germinal center, oxyphilic cell metaplasia (Hürthle cells), atrophy, and fibrosis of thyroid follicles were classified as concomitant HT^10^. Grading of PTC was done according to the criteria of the World Health Organization Classification of Tumors^18^, while staging was done according to the TNM staging system^19^. This study was approved by the Institutional Review Board of Faculty of Medicine Zagazig University (IRB, No. 5673).

### Immunohistochemical staining

The sections (4–5 μm) obtained from paraffin blocks were dewaxed, rehydrated, and placed in 0.5% hydrogen peroxide in methanol for 10 min to block endogenous peroxidase activity. Antigen retrieval was performed by incubation in 0.01 citrate buffer for 5 min in a pressure cooker. The sections were exposed to a primary antibody for 60 min at room temperature. The streptavidin–biotin-peroxidase method was performed using two primary antibodies with dilution 1:500 for anti-FoxP3 mouse monoclonal antibody (clone 236A/E7; Abcam, Cambridge, UK) and dilution 1:100 for anti-CK-19 polyclonal antibody, (Dako Cytomation). Diaminobenzidine (DAB) was used as a chromogen to visualize the immune reaction. CK19 immunostaining accepted positive when there was cytoplasmic or membranous staining and FOXP3 accepted positive when there was a distinct nuclear or cytoplasmic staining^16,20^.

Tonsil and skin were used as a positive control for FOXP3 and CK19 respectively, while the negative control for both markers was done by ignoring the primary antibody.

### Evaluation of immunohistochemical staining

The expression of the two markers was scored using a semi-quantitative method in areas of PTC and also analyzed in surrounding non-neoplastic tissue by two independent pathologists (TI, SB). For FOXP3. The distribution of positive cells was estimated according to the following: 0 = no stained cells; 1 = up to 10% stained cells; 2 = 10–30% stained cells; and 3 ≥ 30% stained cells. Simply, the slides with zero scores were considered negative, and the slides with total scores from one up to three were considered positive^20^.

And for CK19; immunoreactivity was considered negative if less than 10% of stained cells, focal (+: < 25%), positive (++: 25 up to 50%) or diffuse (+++: > 50%), which is according to the distribution of the reaction^16^.

### Statistics

- The collected data were computerized and statistically analyzed using SPSS 22.0 for Windows; SPSS Inc. Chicago, Illinois, USA) and MedCalc windows (MedCalc Software bvba 13, Ostend, Belgium).
- Data were tested for normal distribution using the Shapiro Walk test.
- Qualitative data were represented as frequencies and relative percentages.
- Chi-square test (χ^2^) and Fisher exact were used to calculate the difference between qualitative variables as indicated.
- Survival analysis: Overall survival (OS) was calculated as the time from diagnosis to death or the most recent follow-up contact (censored). Disease-free survival (DFS) was calculated as the time from date of surgical treatment to date of local recurrence or distant metastasis or the most recent follow-up contact that a patient was known as relapse-free or the end date of the study. Stratification of OS and DFS was done according to immunohistochemical markers. Kaplan and Meier's method used to estimate overall and Progression-free survival and log-rank test compared survival curves
- All statistical comparisons were two-tailed with a significance level of p-value ≤ 0.05 indicates significant, p < 0.001 indicates a highly significant difference while p > 0.05 indicates a non-significant difference.

## Results

### Patients' characteristics

Among the studied 80 thyroid biopsies that were obtained from patients with PTC, 62.5% cases were above 45 years, 72.5% were females, 80% were PTC without HT and 20% were PTC with HT. The 80 cases of PTC were classified into 52 cases of classic (or conventional) PTC and 28 cases of follicular subtype PTC (FVPCs). As regards surrounding non-neoplastic tissue, it was normal in 42.5%, HT in 20%, colloid goiter in 17.5%, chronic lymphocytic thyroiditis in 15%, and thyrotoxic hyperplasia in 5% of cases. The LN and distant metastasis are present in 32.5% and 5% patients respectively. 30%of patients received one dose of radioactive I^131^, while 42.5% of patients received more than one dose. Only 27.5% of patients did not receive I^131^ doses as they were of low risk and were on follow up. All patients’ clinicopathologic characteristics are presented in Table 1.

**Table 1:** The clinicopathological features the whole PTC patients (N = 80).

Total N = 80	No	%
**Age (years)**		
≤ 45	30	37.5
> 45	50	62.5
**Sex**		
M	22	27.5
F	58	72.5
**Pathology**		
PTC	64	80.0
PTC with HT	16	20.0
**Path subtype**		
Follicular variant	24	30.0
Classic variant	40	50.0
Follicular variant with HT	4	5.0
Classic variant with HT	12	15.0
**Extracapsular extension**		
No	62	77.5
Yes	18	22.5
**Stage**		
I–II	54	67.5
III–IVA–IVB	22	27.5
IVC	4	5
**Multifocality**		
No	58	72.5
Yes	22	27.5
**Non-neoplastic thyroid tissue**		
Normal	34	42.5
Colloidal	14	17.5
Toxic	4	5.0
Lymphocytic	12	15.0
HT	16	20.0
**LN metastasis (N)**		
No	54	67.5
N1	26	32.5
**T size (cm)**		
≤ 2	50	62.5
> 2	30	37.5
**M**		
M0	76	95.0
M1	4	5.0
**Bilaterality**		
No	68	85.0
Yes	12	15.0
**Number of I**^**131**^** doses**		
No doses	22	27.5
1	24	30
> 1	34	42.5
**Cumulative radioiodine dose (mCI)**		
< 200	38	47.5
≥ 200–400	6	7.5
> 400–600	8	10
> 600–800	4	5
> 800–1000	2	2.5

### Comparison of clinicopathological factors in PTC patients with and without HT (Table 2)

**Table 2:** The clinicopathological differences between Papillary thyroid carcinoma and Papillary thyroid carcinoma with Hashimoto’s thyroiditis (N = 80).

	PTC		PTC with HT		p-value
	N = 64		N = 16		
Age (years)					
≤ 40	24	37.5%	6	37.5%	1
> 40	40	62.5%	10	62.5%	
Sex					
M	20	31.3%	2	12.5%	0.288
F	44	68.8%	14	87.5%	
Pathological subtypes					
Follicular variant	24	37.5%	0	0.0%	< 0.001
Classic variant	40	62.5%	0	0.0%	
Follicular V. with HT	0	0.0%	4	25.0%	
Classic V. with HT	0	0.0%	12	75.0%	
Extrathyroidal extension					
No	46	71.9%	16	100.0%	0.088
Yes	18	28.1%	0	0.0%	
Stage					
I–II	44	68.8%	10	62.5%	0.406
III–IVA–IVB	16	25.0%	6	37.5%	
IVC	4	6.3%	0	0.0%	
Multifocality					
No	46	71.9%	12	75.0%	0.859
Yes	18	28.1%	4	25.0%	
LN metastasis					
No	42	65.6%	12	75.0%	0.613
Yes	22	34.4%	4	25.0%	
Tumor size (cm)					
≤ 2	38	59.4%	12	75.0%	0.414
> 2	26	40.6%	4	25.0%	
Distant metastasis					
No	60	93.8%	16	100.0%	0.468
Yes	4	6.3%	0	0.0%	
FOXP3 immunostaining					
Negative	36	56.3%	8	50.0%	0.751
Positive	28	43.8%	8	50.0%	
CK19 immunostaining					
Negative	14	21.9%	2	12.5%	0.553
Positive	50	78.1%	14	87.5%	
Bilaterality					
No	52	81.3%	16	100.0%	0.184
Yes	12	18.8%	0	0.0%	
Local recurrence					
No	60	93.8%	16	100.0%	0.468
Yes	4	6.3%	0	0.0%	
Death					
No	58	90.6%	16	100.0%	0.368
Yes	6	9.4%	0	0.0%	
Progression					
No	56	87.5%	16	100.0%	0.292
Yes	8	12.5%	0	0.0%	
Number of I^131^ doses					
No doses	22	34.4%	0	0.0%	0.042
One dose	14	21.9%	10	62.5%	
More than one	28	43.8%	6	37.5%	

**Figure 1:**
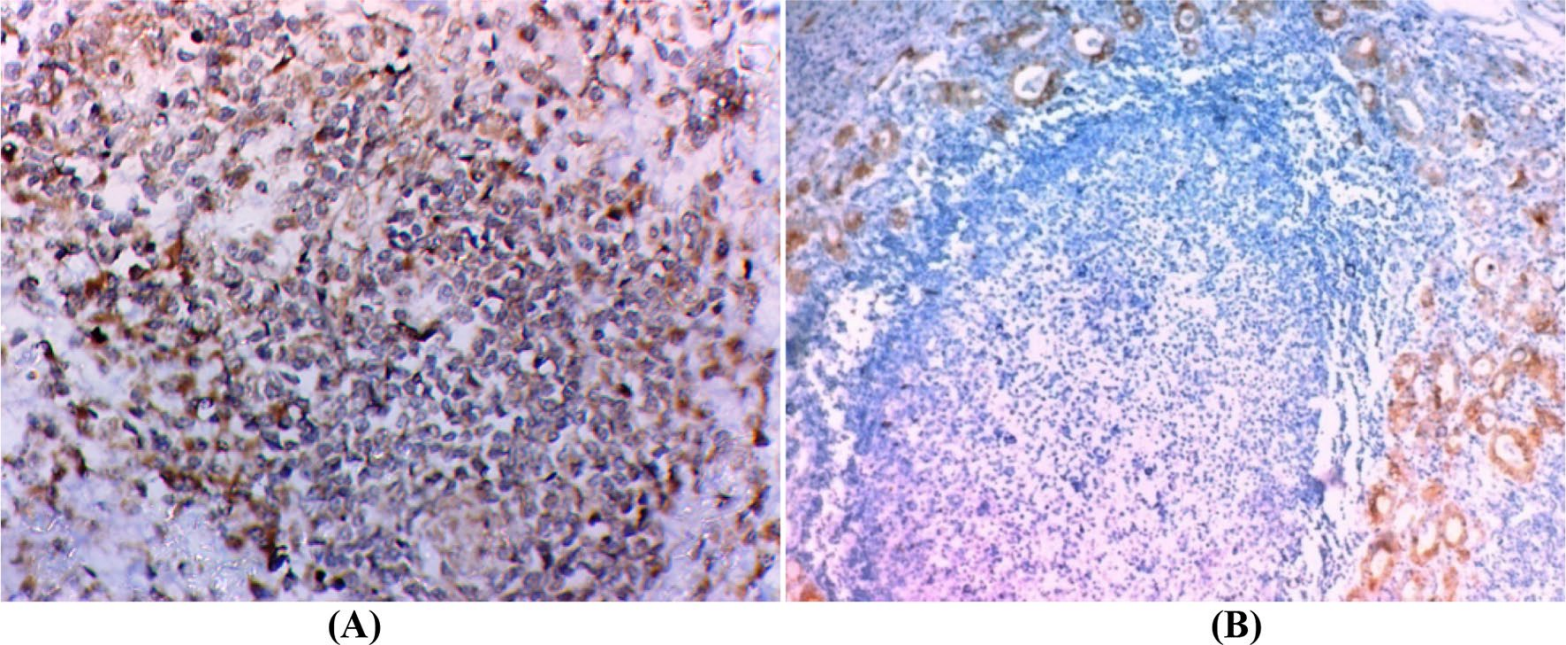
Representative samples of Hashimoto thyroiditis. (**A**) Showing positive nuclear FOXP3 immunostaining in infiltrating lymphocyte (X400). (**B**) Showing positive cytoplasmic CK19 immunostaining in oxyphil cells (× 200).

The current study included 64 cases (80%) diagnosed PTC and 16 cases (20%) diagnosed PTC with concomitant HT (75% of them were classic subtype). On comparing clinicopathological factors between the two groups, although the differences were statistically insignificant patients with PTC and HT tented to have a lower percentage of extrathyroidal extension, advanced stage, lymph node and distant metastasis (negative prognostic variables). There was no statistically significant difference as regard two markers expression between the two groups. Follow up of both groups showed that patients PTC with HT had 100% no local recurrence, deaths, and progressions but the difference was insignificant. As regards to radioactive iodine therapy, there was a statistically significant difference between the two groups (Fig. 1).

### Results of immunohistochemical expression of FOXP3

**Figure 2:**
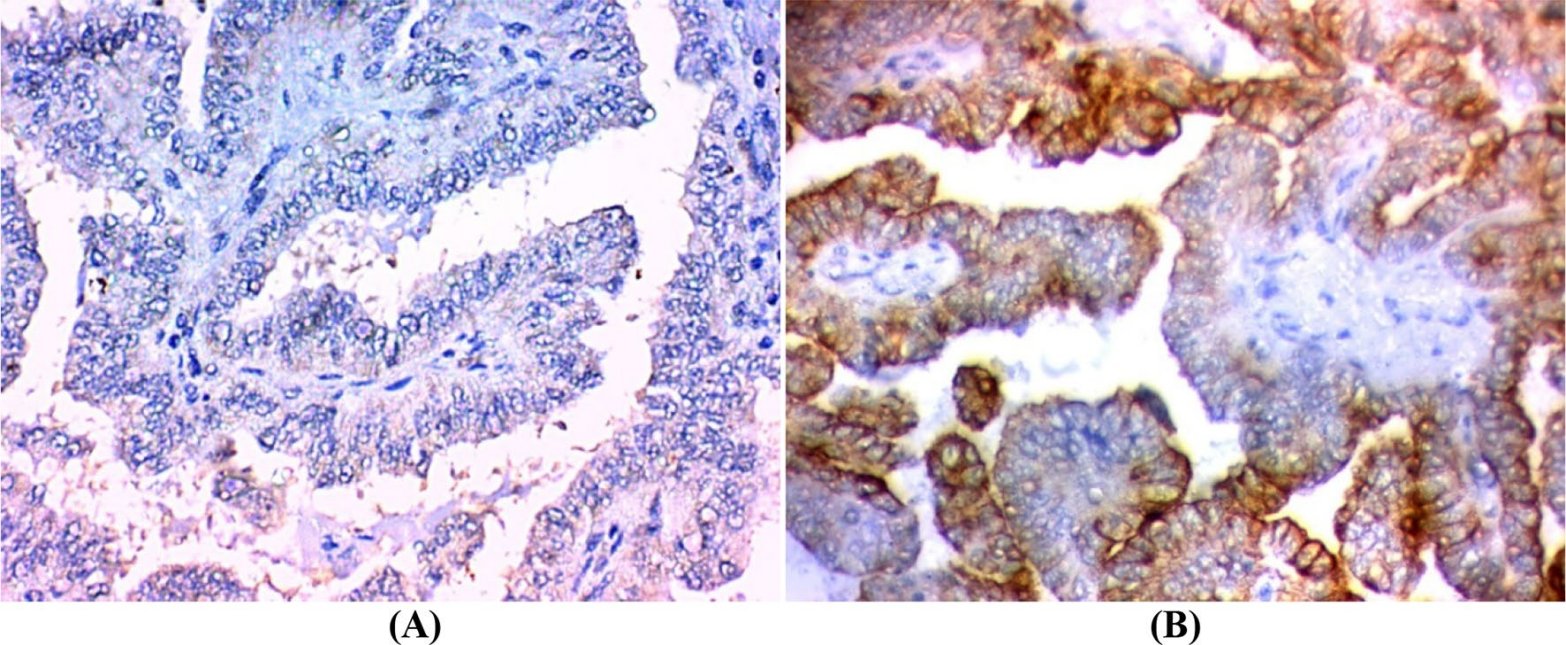
Classic variant of papillary thyroid carcinoma. (**A**) FOXP3 nuclear and cytoplasmic immunostaining (× 200). (**B**) Diffuse CK19 cytoplasmic immunostaining (× 200).

**Figure 3:**
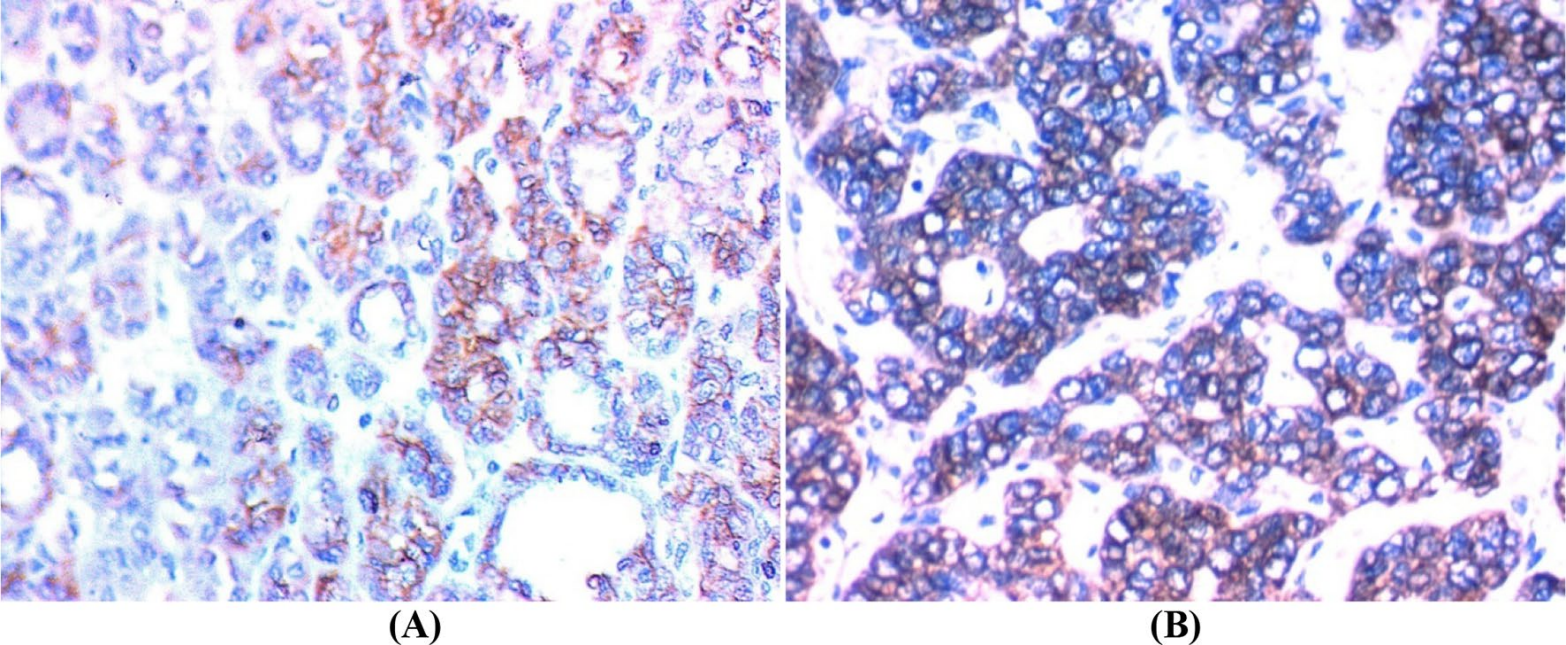
Follicular variant of papillary thyroid carcinoma, (**A**) positive FOXP3 cytoplasmic immunostaining (× 200). (**B**) Positive cytoplasmic and membranous CK19 immunostaining (× 400).

The clinicopathological characteristics of patients with PTC and their association with FOXP3 expression are presented in Table 3. The neoplastic cells showed nuclear and or cytoplasmic FOXP3 immunoreactivity. All positive samples showed weak to moderate FOXP3 expression (Figs. 2A, 3A).

**Table 3:** Association of clinic-pathological features with marker expression in the studied PTC (N = 80).

	FOXP3				p	CK19				p
	Negative N = 44		Positive N = 36			Negative N = 16		Positive N = 64		
	N	%	N	%		N	%	N	%	
**Age (years)**										
≤ 45	28	63.6	2	5.6	< 0.001	6	37.5	24	37.5	1
> 45	16	36.4	34	94.4		10	62.5	40	62.5	
**Sex**										
M	4	9.1	18	50.0	0.004	6	37.5	16	25.0	0.479
F	40	90.9	18	50.0		10	62.5	48	75.0	
**Pathology**										
PTC	34	77.3	30	83.3	0.634	14	87.5	50	78.1	0.553
PTC with HT	10	22.7	6	16.7		2	12.5	14	21.9	
**Path subtype**										
Follicular	8	18.2	16	44.4	0.307	8	50.0	16	25.0	0.234
Classic	28	63.6	12	33.3		6	37.5	34	53.1	
Follicular with HT	2	4.5	2	5.6		2	12.5	2	3.1	
Classic with HT	6	13.6	6	16.7		0	0.0	12	18.8	
**Extrathyroid Ext**										
No	44	100.0	18	50.0	< 0.001	10	62.5	52	81.3	0.256
Yes	0	0.0	18	50.0		6	37.5	12	18.8	
**Stage**										
I–II	44	100	10	27.8	< 0.001	10	62.5	44	68.7	0.592
III–IVA–IVB	0	0.0	22	61.1		4	25	18	28.1	
IVC	0	0.0	4	11.1		2	12.5	2	3.1	
**Multifocality**										
No	42	95.5	16	44.4	< 0.001	14	87.5	44	68.8	0.288
Yes	2	4.5	20	55.6		2	12.5	20	31.3	
**Non-neoplastic thyroid tissue**										
Normal	26	59.1	8	22.2	0.063	10	62.5	24	37.5	0.753
Colloidal	4	9.1	10	27.8		2	12.5	12	18.8	
Toxic	0	0.0	4	11.1		0	0.0	4	6.3	
Lymphocytic	4	9.1	8	22.2		2	12.5	10	15.6	
HT	10	22.7	6	16.7		2	12.5	14	21.9	
**LN metastasis**										
No	42	95.5	12	33.3	< 0.001	10	62.5	44	68.8	0.736
N1	2	4.5	24	66.7		6	37.5	20	31.3	
**T**										
T1–T2	44	100	18	49.9	< 0.002	10	62.5	52	81.1	0.262
T3–T4	0	0.0	18	49.9		6	37.5	12	18.57	
**M**										
No	44	100.0	32	88.9	0.109	14	87.5	62	96.9	0.277
Yes	0	0.0	4	11.1		2	12.5	2	3.1	
**Bilaterality**										
No	42	95.5	26	72.2	0.041	14	87.5	54	84.4	0.825
Yes	2	4.5	10	27.8		2	12.5	10	15.6	
**Number of iodine doses**										
No doses	22	50	0	0.0	0.001	6	37.55	16	25	0.888
One dose	14	31.8	10	27.7		4	25	20	31.2	
More than one	8	18.18	26	72.2		6	37.5	28	43.7	
**Cumulative iodine dose (mCI)**										
< 200	30	68.1	8	22.2	0.001	8	50	30	16.8	0.673
≥ 200–400	4	9.09	2	5.5		2	12.5	4	6.25	
> 400–600	2	4.5	6	16.6		4	25	4	6.25	
> 600–800	0	0.0	4	11.1		2	12.5	2	3.12	
> 800–1000	0	0.0	2	5.5		0	0.0	2	3.12

In PTC, the positiveFOXP3 expression was more in female patients for more than 45 years with a statistically significant difference (p = 0.004 and p < 0.001, respectively).

Besides the female gender, FOXP3 expression was related to a series of poor clinicopathological features like large tumor size, higher tumor stage, presence of lymph node and distant metastasis, multifocality, and extracapsular extension with a highly statistically significant difference (p < 0.001) and bilateral tumors (p = 0.041).

As regards pathological variants of PTC, in classic subtype without associated HTFOXP3 expression was detected in 33.3% while it was detected in 16.7% in a classic variant with HT. In studied cases of follicular variant without associated HT, FOXP3 expression was detected in 44.4% while it was detected in a 5.6% follicular variant with HT with no significance statistically.

There is no statistically significant difference between FOXP3 expression in PTC and surrounding non-neoplastic thyroid tissue (p value = 0.063). PTC with associated HT showed FOXP3+ lymphocytes in the lymphocytic infiltrate of HT indicating the presence of regulatory T lymphocytes (Treg) (Fig. 1A).

As regards the expression of FOXP3 and radioiodine therapy: The positive expression of FOXP3 in PTC cells was associated significantly with I^131^ doses more than one dose and cumulative radioiodine doses (p < 0.001). In the current study, FOXP3 expression in PTC was associated with negative prognostic factors such as; large tumor size, bilaterality, multifocality, extracapsular invasion, and the presence of lymph node and distant metastasis, which may have a specific impact on radioiodine sensitivity.

### Results of immunohistochemical expression of CK19

Among the studied cases, cytoplasmic CK19 expression was detected in 80%of papillary thyroid carcinoma (PTC) (Figs. 2B, 3B). No significant differences in CK19 expression and different clinicopathological variables (Table 3). In cases of PTC with associated HT, there is a positive cytoplasmic expression of CK19 in Hurthle (oxphill) cells. (Fig. 1B).

Neither number of radioactive I^131^ doses nor its cumulative doses showed significant association with CK19 expression.

No significant relationship between FOXP3 expression and CK19 expression in the studied cases of PTC (p-value = 0.634) (Table 5s).

### Clinical outcomes

**Figure 4:**
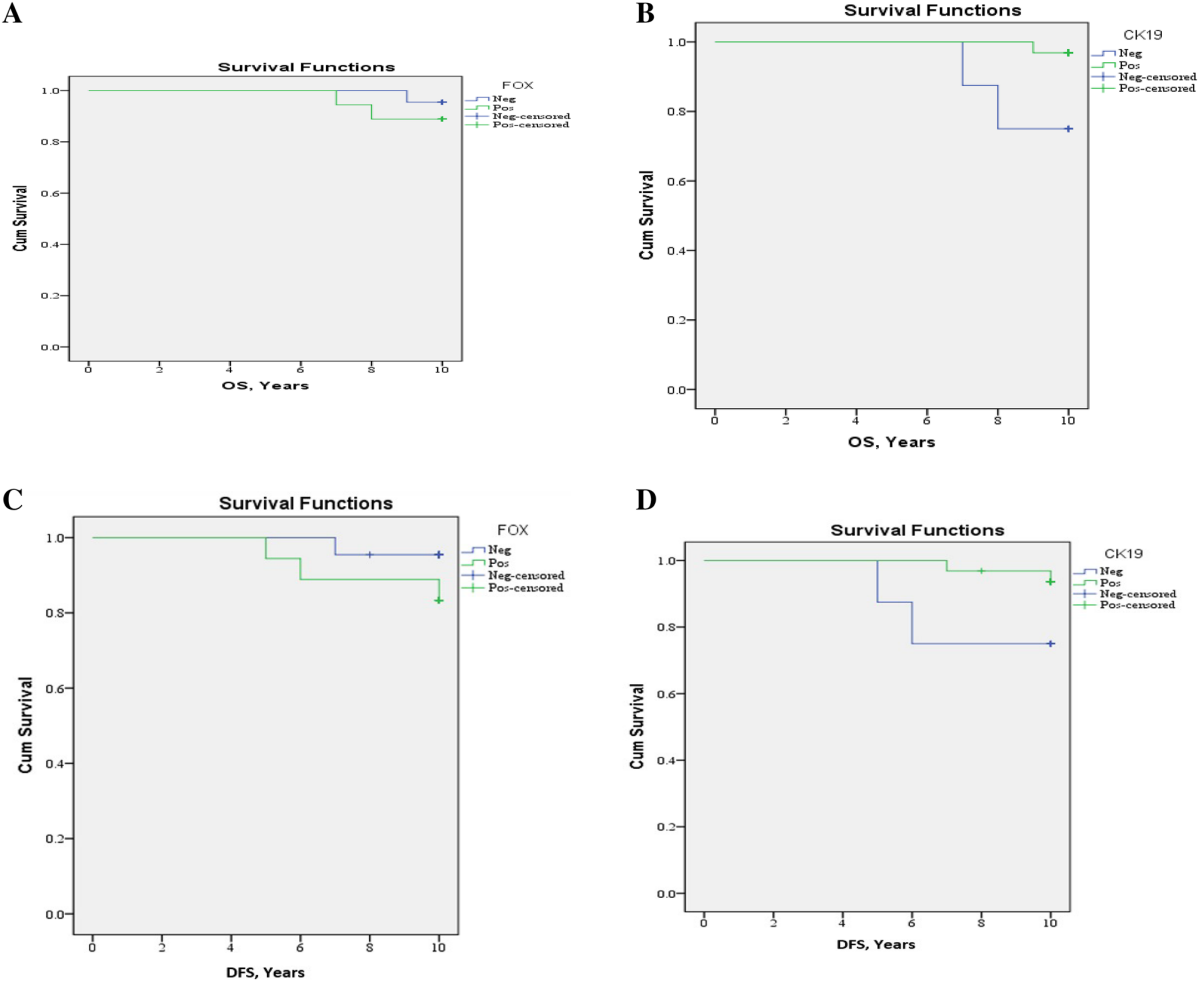
(**A**) 10-year Overall Survival of the studied group as regard FOXP3 expression. (**B**) 10-year Overall Survival of the studied group as regard CK19 expression. (**C**) 10-year Disease-Free Survival as regard FOXP3 expression. (**D**) 10-year Disease-Free Survival as regard CK19 expression.

After a median follow-up period of 10 years with a range (7–10) year,10 years OAS was 92.5% with 95% CI (9.7–10), Mean ± SE (9.9 ± 0.1) 0.10 year DFS was 89.2% with 95% CI (9.3–10.1), Mean ± SE (9.7 ± 0.2) (Fig. 4A–D). No significant association was found between the expression of FOXP3 and distant metastasis, local recurrence, and death. While there was a significant correlation between CK19 expression and death (p value = 0.036) (Table 4). There was a significant correlation between CK19 expression and 10 year overall survival (p value = 0.027).

**Table 4:** Clinical outcome of patients concerning markers expression.

Clinical outcome	Total N = 80		FOXP3				p	CK19				p
			Negative N = 44		Positive N = 36			Negative N = 16		Positive N = 64		
	N	%	N	%	N	%		N	%	N	%	
**Metastasis**												
No	72	90.0	42	95.5	30	83.3	0.204	12	75.0	60	93.8	0.114
Yes	8	10.0	2	4.5	6	16.7		4	25.0	4	6.3	
**Local recurrences**												
No	76	95.0	42	95.5	34	94.4	0.884	16	100.0	60	93.8	0.468
Yes	4	5.0	2	4.5	2	5.6		0	0.0	4	6.3	
**Death**												
No	74	92.5	42	95.5	32	88.9	0.433	12	75.0	62	96.9	0.036
Yes	6	7.5	2	4.5	4	11.1		4	25.0	2	3.1	

## Discussion

Thyroid carcinoma is the commonest malignancy of the thyroid gland, it is about 1% of all malignant tumors, and PTC is the commonest form about 80–90% and their etiology is still unclear^21^. PTC has an excellent prognosis with high survival rates, but 10%–15% of cases have advanced disease with lymph node metastasis at the time of diagnosis. These patients require new therapeutic modalities besides traditional treatments like combined surgical and radioiodine therapy to reach a definitive cure^22^.

In this study, we examined the clinicopathological and prognostic value of immunohistochemical expression of FOXP3 and CK19 in PTC suggesting a role in the pathogenesis of PTC and if there was a difference in their expression in concomitant HT and their correlation with patients' outcome and survival.

The coexistence between PTC and HT has been reported in many studies, but it is still controversial. In this work, the incidence of PTC associated with HT was 20%. This finding nearly the same as Ahn et al., who reported that 21.6% of PTC had concurrent HT^1^. However, Girardi et al., found the association between PTC and HT was 35.4%^10^. The variation in the incidence between studies; may be attributed to the differences in pathological interpretation of HT. Occasionally nonspecific lymphocytic infiltration represents the response of the immune system to tumor misdiagnosed as HT. However, we emphasize that the expression of PTC related marker (FOXP3) by HT infiltrating lymphocyte, point toward a relationship between the development of HT and PTC (the multistep process in autoimmune inflammatory disease).

In this study, PTC with coexisting HT was associated with good prognostic variables such as younger age, smaller tumor size, unifocal tumor, a lower percentage of lymph node and distant metastasis in comparison to PTC alone. But the relationship was statistically insignificant may be due to small sample size. These results were in line with Ahn et al., who reported that patients with PTC and HT associated with a good prognosis^1^. Marotta et al. reported that concomitant HT can be considered as a protective shield against PTC progression despite the presence of BRAF mutation which is one of the bad prognostic factors of PTC^23^. As regards clinical follow-up, PTC patients with concomitant HT tend to have better overall survival rates but not reach the significance level, these findings were in line with Ahn et al.^1^. Marotta et al. demonstrated that concomitant HT is considered an independent prognostic variable in intrathyroidal PTC, but failed to improve prognostic specificity^24^.

Previously, FOXP3 was considered to be expressed only in the regulatory T cells, but now it has been found in multiple types of malignant cells^25,26^. Tumors with positiveFOXP3are more liable to invasion and metastasis through induction of the secretion of some cytokines which have immunosuppressive functions such as TGF-β1 and IL-10^27^. This may represent an example of molecular simulation and could represent a masked method of the escape of malignant cells from the immune system^28^.

Among the studied PTC, FOXP3 expression was detected in 45% of the cases, this finding is nearly similar to the finding obtained by Ugolini et al., who reported FOXP3 expression in43% of the studied PTC^22^. Other related studies reported a higher incidence of FOXP3 expressions among the studied PTC such as Junior et al., who reported FOXP3 positivity in 72.4% of the studied PTC^12^, Cunha et al., detected FOXP3 expression in 91.9% of malignant differentiated thyroid carcinomas and 71% of benign thyroid lesions^20^, Ma et al., reported FOXP3 expression in 74% of the studied PTC, while benign thyroid lesions were lacking FOXP3 expression^28^, and Zhang et al., found FOXP3 positivity in all PTC and few of non-malignant follicular lesions with statistically significant difference^29^.

These differences in the expression of FOXP3 in PTC may be due to the usage of different primary antibody clones, different immunohistochemical techniques, a different method of interpretation of markers positivity, and may be due to different cohort number.

Our results showed that FOXP3 tends to be more expressed in PTC cases with large tumor size, advanced tumor stage, presence of lymph node metastasis, and extracapsular extension with a highly statistically significant difference (p < 0.001).These results in line with Ugolini et al., who found in their study thatFOXP3 immunostaining was associated with finding distant spread and extension outside the thyroid gland^22^.

Cunha et al. demonstrated the absence of association between FOXP3 expression and clinical or pathological features of tumor progression or increased patient survival. While lymphocytes that are positive toFOXP3 were more found in smaller tumors (less than 2 cm), tumors without the presence of infiltration outside the thyroid gland, and cases who have concurrent chronic lymphocytic inflammation of the thyroid and concluded that FOXP3 expression had an important impact on the aggressiveness of the tumor is low-grade thyroid cancer, especially in cases with strong nuclear staining^20^.

French et al. found that the presence of regulatory T cell infiltration was ultimately correlated with the occurrence of metastasis to lymph node^30^. Liu et al. reported that an increased level of FOXP3+ Treg both in the tumor tissue and in the serum of the patient was correlated to extrathyroidal invasion and lymph node spread and supposed that increased Treg cells are associated with poor outcome of the malignancy^14^. It is difficult to compare our results with those obtained by French et al. and Liu et al. because they not evaluate FoxP3 expression in malignant tissue but investigated the expression of FOXP3 in regulatory T cells and lymphocytes infiltrating the PTC.

Our results showed there is a lack of statistically significant difference between FOXP3 expression in PTC and surrounding non-neoplastic thyroid tissue (p value = 0.063). Cases of PTC with concomitant Hashimoto thyroiditis showed scattered FOXP3+ lymphocytes in the lymphocytic infiltrate of Hashimoto thyroiditis indicating the presence of regulatory T lymphocytes (Treg). Yang et al. demonstrated that there was a decreased expression of FOXP3and improper Treg function in patients with HT may be due to abnormal acetylation of FOXP3^31^.

In this work FOXP3 expression detected in 83.3% of PTC without HT and in 16.7% of PTC with associated HT. The results showed that there were no significant differences (p = 0.634), evidencing that expression of FOXP3 in papillary carcinoma is independent of the coexistence of HT.

These results are confirmed by Junior et al., who revealed that the absence of a significant difference in FOXP3 expression in PTC with or without HT. This finding suggests that FOXP3 expression is not related to the presence of HT but the process of malignant transformation should be responsible for the abnormal FOXP3 expression detected in PTC cells^12^.

CK19 expression was detected in 80%of our studied PTC. Calangiu et al. found that CK19 expression in 84.6% of PTC^32^. Divani et al. demonstrated that Ck19 showed positive expression in all PTC cases with strong diffuse cytoplasmic reactivity and concluded that it is a sensitive marker for PTC with diffuse cytoplasmic positivity^33^. These results were the same as that of Alshenawy who found that CK19 expression detected in all cases of PTC (100% of classic PTC and 100%of follicular variant PTC^16^.

Kaliszewski et al., found in their study which was done on the classic variant of papillary thyroid carcinoma that cytoplasmic CK19 positivity was detected in 95.3% of the patients^34^.

Huang et al. reported that 96.7% of PTC had positiveCK19 expression, 95.3% of PTMC (papillary thyroid micro-carcinoma), 5.9% of nodular goiter, and 3.6% of HT. Thus, CK19 expression is indicated to be a valuable immunohistochemical marker in the diagnosis of cancer thyroid^35^.

Our study showed no significant differences in CK19 expression and different clinicopathological variables, this was in agreement with previous related studies^32,34^.

In cases of PTC with associated HT, there is a positive cytoplasmic expression of CK19 in Hurthle (oxphill) cells. These results are confirmed by Ma et al., who reported that all studied HT showed scattered CK19 expression in Hurthle cells which were near or adherent to the lymphoid follicles and displaying intense eosinophilic metaplasia^36^.

Our results detected the expression of PTC-associated proteins CK19 and FOXP3 in HT, this supports the hypothesis that epithelial changes of thyroid follicles in HT represent a multistep process of autoimmune inflammatory disease ending by the evolution of PTC, therefore appropriate follow up of these cases are needed. This conclusion was supported by previous related studies^36,37^.

Our study showed no significant relationship between FOXP3 expression and CK19 expression in the studied cases of PTC (p-value = 0.634). To our knowledge we are the first study evaluated the relationship between the two markers in PTC in the Egyptian population, so further studies on a wide scale of patients are recommended.

This work showed no significant correlation was detected between the expression of FOXP3 and metastasis, local relapse, and death. Also, no association was found between the expression of FOXP3 and overall survival and disease-free survival of PTC cases. This finding was in agreement with Ugolini et al., who stated that no correlation was detected between the expression of FOXP3 and OS or DFS^22^.

The expression of FoxP3was not associated with overall survival may be due to the nature of PTC that explains these contradictory data. PTC is characterized by a good prognosis with high survival rates of over 20 years whileFOXP3 usually associated with poor prognostic variables^38^.

Ladoire et al. investigated the expression of HER2- in breast carcinomas, stated that FoxP3 expression can be used as a separate prognostic factor for increased both relapse-free and overall survival of these patients^39^.

On the other hand, Merlo et al. found an inverse relationship between expression of FoxP3 in malignant cells and patient survival, they also demonstrated the presence of a significant relationship between FOXP3 expression and lymph node spread, concluding that FOXP3 expression is considered as a bad prognostic marker in breast cancer^40^.

In our study, there was a significant correlation between CK19 expression and both of death and 10-year overall survival (p value = 0.036 and 0.027, respectively). Kaliszewski et al., a study that found significant CK19 overexpression in the patients with better survival and no recurrence detected during follow up period, and concluded that CK19 overexpression was associated with good postsurgical prognosis without recurrent disease and suggested that decreased CK19 expression of maybe an indication of recurrent disease^34^. Liu et al., examined the correlation between CK19 and aggressive behavior of PTC stated that CK19 expression was significantly correlated to total tumor size and hence related to PTC progression^41^. This discrepancy may be explained by geographical differences and differences in sample size between different studies.

Our study showed a significant correlation between PTC alone and PTC with concomitant HT and FOXP3 expression with the number of radioactive iodine doses and the cumulative radioiodine doses (p = 0.042 and < 0.001, respectively).This may be attributed to the association between FOXP3 expression and unfavorable prognostic factors that have a specific impact on radioiodine sensitivity. Radioactive iodine cumulative doses are related directly to the severity of the case concerning the pathology and the response to the ablation dose. This may explain the indirect relationship between FOXP3 overexpression and cumulative iodine therapy.

While there was no significant correlation between CK19 expression and iodine therapy. This finding is consistent with Ugolini et al. study that found a significant association between positive FOXP3 and the cumulative radioactive iodine doses^22^. Riesco-Eizaguirre et al., stated that FOXP3 expression in malignant cells is associated with radioiodine treatment resistance and added it should be recalled that BRAF mutations are frequent in PTC and may be associated with the loss of the sodium/iodide symporter. This event is associated with radioiodine-refractory disease^42^.

## Conclusion

HT represents a step in the process of autoimmune inflammatory disease ending by the evolution of PTC, therefore appropriate follow up of these cases is needed. PTC with concomitant HT associated with a better prognostic course. Our study showed that FOXP3 tends to be more expressed in PTC cases with worse prognostic variables. So, we predict that FOXP3 will become a new prognostic and a novel therapeutic approach for PTC. Discovering new treatment modalities against FOXP3 is very promising because it can decrease the suppression of immune function, and may improve patients with cancers that are resistant to radioiodine therapy. There was a significant relationship between CK19 expression and10 year overall survival.

## Supplementary information

Supplementary information.
