# Supplementary material for: Clinicopathological characterization and prognostic implication of FOXP3 and CK19 expression in papillary thyroid carcinoma and concomitant Hashimoto's thyroiditis
Source: Sci Rep. 2020 Jun 30;10:10651. doi: 10.1038/s41598-020-67615-0 (PMC7326975; doi:10.1038/s41598-020-67615-0)
Supplement: Supplementary file 1 — Supplementary information. [file 41598_2020_67615_MOESM1_ESM.docx]

Clinicopathological characterization and Prognostic implication of FOXP3 and CK19 expression in papillary thyroid carcinoma and concomitant Hashimoto's thyroiditis

**Authors:**

**Salem Youssef Mohamed^4^, Taiseer R. Ibrahim^1^, Samah S. Elbasateeny^1^, Lobna A. Abdelaziz^2^, Shaimaa Farouk2, Mahmoud Abdou Yassin^3^, Ahmed Embaby^4^**

1-Pathology Department, Faculty of Medicine, Zagazig University, Egypt.

2-Clinical Oncology and Nuclear Medicine Department, Faculty of Medicine, Zagazig University, Egypt.

3-General Surgery Department, Faculty of Medicine, Zagazig University, Egypt.

4-Internal Medicine Department, Faculty of Medicine, Zagazig University, Egypt.

Corresponding author: Salem Youssef Mohamed

Assistant professor of internal medicine, gastroenterology and Hepatology unit, faculty of medicine, Zagazig University, Egypt.

Mail: [salemyousefmohamed@gmail.com](mailto:salemyousefmohamed@gmail.com)

Cell phone: +201147805292.

ORCID: 0000-0003-2917-4293.

**Supplementary material**

**Supplementary Table5 : markers co-expression in the studied PTC**

|  | | FOXP3 | | | | Total  N=80 | | P |
| --- | --- | --- | --- | --- | --- | --- | --- | --- |
|  |  | Negative  N=44 | | Positive  N=36 | |  |  |  |
|  |  | N | % | N | % | N | % |  |
| CK19 | Negative | 10 | 22.7% | 6 | 16.7% | 16 | 20.0% | 0.634 |
|  | Positive | 34 | 77.3% | 30 | 83.3% | 64 | 80.0% |  |

**Supplementary Table 6: Mean survival time and survival rates concerning each marker**

| Markers | | Survival Rate% | Survival Time (Years) | | | P | |
| --- | --- | --- | --- | --- | --- | --- | --- |
|  |  |  | Mean ± SE | (95% CI) | |  |  |
| 10-year Overall Survival | | | | | | | |
| Overall | | 92.50% | 9.9 ± 0.1 | (9.7-10) | |  | |
| FOXP3 | Negative | 95.50% | 10 ± 0 | (9.9-10) | | 0.42 | |
|  | Positive | 88.90% | 9.7 ± 0.2 | (9.4-10.1) | |  |  |
| CK19 | Negative | 75% | 9.4 ± 0.4 | (8.6-10.1) | | 0.027 | |
|  | Positive | 96.90% | 10 ± 0 | (9.9-10) | |  |  |
| 10-year Disease-Free Survival | | | | | | | |
| Overall | | 89.20% | 9.7 ± 0.2 | | (9.3-10.1) | |  |
| FOXP3 | Negative | 95.50% | 9.9 ± 0.1 | | (9.6-10.1) | | 0.208 |
|  | Positive | 83.30% | 9.5 ± 0.4 | | (8.7-10.3) | |  |
| CK19 | Negative | 75% | 8.9 ± 0.7 | | (7.5-10.2) | | 0.089 |
|  | Positive | 93.60% | 9.9 ± 0.1 | | (9.7-10.2) | |  |
